# Multidetector Computed Tomographic Angiography (MDCTA) for Penetrating Neck Injuries

**DOI:** 10.5041/RMMJ.10084

**Published:** 2012-07-31

**Authors:** Jason Pasley, Regan J. Berg, Kenji Inaba

**Affiliations:** Los Angeles County Medical Center—University of Southern California, Division of Trauma Surgery and Surgical Critical Care, Los Angeles, California, USA

**Keywords:** Angiography, clinical decision-making, computed tomography, neck injury, penetrating trauma

## Abstract

Evaluation of patients after penetrating neck injury has evolved over time. Previously, location of injury and symptoms were used to determine management. The contemporary management of penetrating neck injuries relies on physical examination. Patients with hard signs of vascular or aerodigestive tract injury require immediate operation, regardless of location of injury. Those with no signs can be observed. For the remainder with soft signs, multidetector computed tomographic angiography (MDCTA) is a highly sensitive and specific screening modality for evaluating the vasculature and aerodigestive structures in the neck. Utilizing MDCTA, the patient can be safely directed towards operative intervention, observation, or further investigation.

## INTRODUCTION

Work-up and management of penetrating cervical injuries have evolved over time. Routine exploration of patients with penetrating neck trauma was popular during World War II; however, due to the highly negative exploration rates and considerable resultant morbidity the paradigm changed.^1^ These concerns led to development of alternative management strategies, which integrated physical exam findings, hemodynamic status, and selective multi-modal diagnostic investigation, influenced by the exact anatomic location of injury.^2^ Although widely adopted, this approach still suffers from a requirement for multiple invasive investigations with significant associated hospital cost and potential morbidity. Most recently, incorporation of multidetector computed tomographic angiography (MDCTA) into the diagnostic armamentarium for penetrating neck trauma has significantly changed management. This examination enables clinicians to rapidly diagnose vascular injury in clinically stable patients and avoid more invasive tests or needless operative exploration. It is rapidly becoming the standard of care for patients with these injuries.

## INITIAL ASSESSMENT OF NECK IN THE TRAUMA PATIENT

Initial management of the patient with penetrating trauma proceeds in accordance with the general principles articulated by the American College of Surgeons Advanced Trauma Life Support (ATLS) course. Airway, breathing, and circulation are evaluated, and immediate life threats addressed. Current assessment of a patient with penetrating neck trauma integrates clinical findings with additional results, in clinically stable patients, from selective diagnostic investigation. Clinical findings have been traditionally classified as “hard” or “soft,” depending on whether obvious evidence of vascular or aerodigestive injury is present. “Hard” signs include active hemorrhage, shock unresponsive to initial fluid therapy, a pulsatile or expanding hematoma, bruit, or thrill, massive hemoptysis or hematemesis, or air bubbling through the wound. Patients presenting with “hard” signs require emergent operative exploration. “Soft” signs include venous oozing, non-expanding or non-pulsatile hematoma, minor hemoptysis, dysphonia, dysphagia, and subcutaneous emphysema. These patients, if they remain clinically stable, are suitable for further investigation. The site of anatomic injury, classified by dividing the neck into three distinct “zones,” has also traditionally been used to determine the need for operative exploration or selective diagnostic investigation. Zone 1 extends from the clavicles to the cricoid cartilage; zone 2, from the cricoid cartilage to the angle of the mandible; and zone 3, from the angle of the mandible to the base of the skull. A presumptive direct relationship between the superficial site of trauma and underlying injury to deeper structures underlies this approach. Zone 2 injuries are the most accessible to direct surgical exploration, whereas wounds in zone 1 and zone 3 are often challenging or inaccessible for surgical intervention and are best served by less-invasive diagnostic and therapeutic approaches. Although commonly utilized, this anatomic distinction can be problematic as an entry wound in zone 2 could easily affect structures in zone 1 or zone 3 without this being immediately apparent from the superficial wound site.

## ROLE OF CONVENTIONAL ANGIOGRAPHY FOR VASCULAR INJURY

Previous to the evolution of MDCTA, conventional catheter-based angiography was the standard technique for evaluation of cervical vasculature injury. Although highly accurate, this technique is limited by logistic considerations as well as its invasive nature. Conventional angiography requires mobilization of a specialized team, composed of both radiologists and non-physician technical staff, a resource often unavailable with the 24-hour coverage required for the continuous care of trauma patients. Even in centers with on-call interventional radiologic teams, the time required to mobilize this group will often preclude their ability to assist with diagnosis and operative planning in all but the most stable of patients. Additionally, conventional angiography is associated with a low but nonetheless significant local complication rate. Dissection, thrombosis, or pseudoaneurysm of the access vessel; formation of an embolizing plaque; or local hematoma may all complicate conventional angiographic access.

## ADVANTAGES OF MDCT ANGIOGRAPHY

MDCTA has emerged as a highly accurate diagnostic modality that avoids the complications of an invasive procedure and can be easily incorporated into the standard care of trauma patients without significant logistical constraints. The patient is taken to the CT scanner right from the trauma bay. The examination is performed by the radiology technician, utilizing pre-existing hardware, software, and contrast injectors. The patient is not under a sterile field, allowing direct monitoring throughout the procedure. MDCTA is rapid, with images obtained in less than 1 minute and easily integrated into the examination of patients with multi-system trauma. Due to the quick nature of the exam, no additional sedation or pain medication is necessary, other than what is clinically indicated. The contrast is run through a peripheral IV, negating significant risk of local complications, and the contrast load is comparable to a typical 4-vessel run-off angiography. The radiation dose is approximately 1200 MGy/cm, with some variation based on body habitus. This level is below that of standard diagnostic screening angiogram DSA.

**Figure 1:**
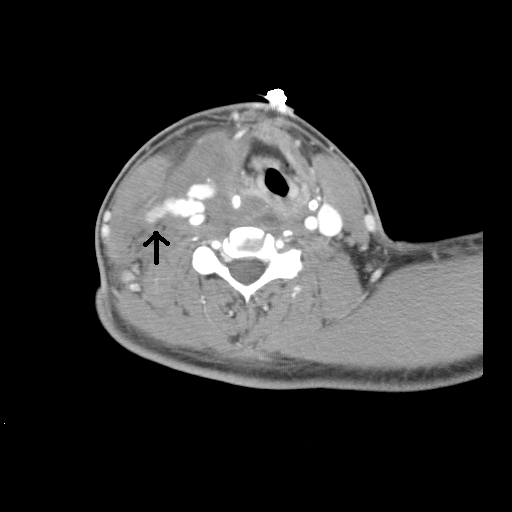
Axial image of the neck with extravasation (arrow) from the right common carotid.

**Figure 2:**
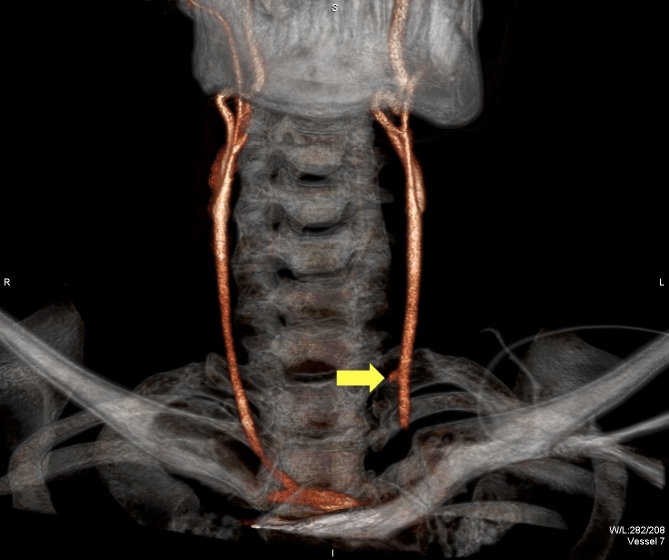
Three-dimensional reconstruction with pseudoaneurysm (arrow) of the common carotid after a stab wound to the neck.

MDCTA images visualize the vascular system as well as aerodigestive structures, soft tissue, and cervical spine, providing efficient and accurate assessment of all potential injuries with this one study (Figure 1). Unlike conventional angiography, MDCTA is more easily interpreted by operating surgeons familiar with evaluating axial images. With the multi-slice detector, high-resolution images can be obtained and three-dimensional reconstructions performed, giving a clear picture of the injured structures, allowing more accurate operative planning (Figure 2).

## DETECTION OF ESOPHAGEAL AND TRACHEAL INJURY WITH CONVENTIONAL STRATEGIES

Evaluation of the aerodigestive tract has traditionally been dependent on multiple, invasive modalities, including flexible fiberoptic laryngoscopy, esophagoscopy, bronchoscopy, and contrast esophagraphy. These methods are time-and resource-consuming, costly, and typically associated with low diagnostic yield when used as screening tools. As MDCTA technology increases in accuracy, the indications for these investigations are being increasingly re-evaluated.

## MDCTA FOR DETECTION OF ESOPHAGEAL AND TRACHEAL INJURY

Esophageal and aerodigestive tract injuries remain relatively rare findings in penetrating neck injuries.^3^ MDCTA allows clinicians to assess the probability of aerodigestive injuries by delineating the missile tract. Patients with tracts remote from key structures are unlikely to have significant injury and can be safely observed. Patients demonstrating concerning missile tracts or additional evidence suggestive of injury can then undergo further directed testing with endoscopy or contrast studies. By utilizing MDCTA as a first-line investigation, patients can be appropriately triaged and further invasive investigation appropriately performed without undue delay.

## TECHNICAL PROTOCOL

At the Los Angeles County—University of Southern California (LAC+USC) Medical Center, the standard MDCTA neck protocol uses the following parameters: 120 kVp, 100 mA to 250 mA (depending on size of the patients, using dose modulation), gantry revolution speed of 0.5 second, beam pitch 0.656, beam collimation of 64 mm × 0.5 mm, variable field of view (depending on patient size), and standard body kernel. A line suitable for power contrast injection (18–20 gauge peripheral IV line in the antecubital fossa or a central venous catheter approved by the manufacturer for power injection) is utilized for injection of 75–100 mL of iohexol iodinated IV contrast material (Omnipaque 350; GE Healthcare, Princeton, NJ) at a rate of 4–5 mL/s, followed by a 40-mL saline flush, all administered by a Medrad power injector (Spectris; Medrad, Indianola, PA). Contrast bolus tracking with a trigger threshold of 180 HU is used with the region of interest placed in the carotid artery at the C2–3 level. Reconstruction with section thickness of 1 mm in the axial, coronal, and sagittal planes is performed and additional post-processing accomplished using a Vitrea reformatting workstation (Vital Images, Plymouth, MN), creating volume renderings, maximum intensity projections, and curved planar reformats as needed.

## PITFALLS AND LIMITATIONS

**Figure 3:**
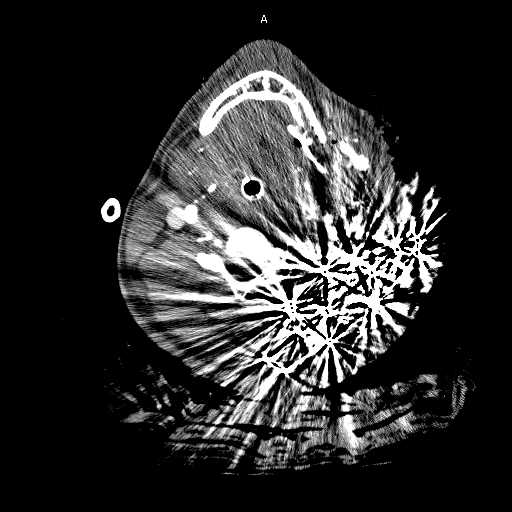
Scatter from shotgun pellets.

Although a highly sensitive and efficient modality for diagnosis of vascular injury, MDCTA evaluation may be compromised by technical limitations. Streak artifacts from retained missiles or shoulders of large patients can potentially mask clinically significant injury. This is particularly problematic in the case of retained missile or shotgun injury (Figure 3). In a large prospective study of 453 patients with penetrating neck trauma, MDCTA was non-diagnostic in 4 patients (1.8%), primarily due to artifact.^3^ Errors in timing of contrast administration and image acquisition can also occur, and image quality may additionally be affected by patient motion. Compared to conventional angiography, the lack of therapeutic capacity may also subject patients with evidence of injury to subsequent repeat contrast administration during a second, interventional procedure. Despite these limitations, MDCTA remains a useful triage and assessment tool to determine who will benefit from further investigation, endovascular treatment, open surgical repair, or observation.

## MANAGEMENT OF PENETRATING NECK TRAUMA AT LAC+USC

MDCTA is integrated into the diagnostic algorithm for neck injury at the LAC+USC Medical Center, used in conjunction with physical exam findings and plain radiographs. Through these modalities patients are selected for observation, operative or endovascular intervention, or further invasive diagnostic testing. Hemodynamic instability or “hard” signs of injury mandate immediate operative exploration. Asymptomatic patients, or those without signs of obvious injury, undergo observation. Patients who are clinically stable but possess “soft” signs of injury are further evaluated with a screening MDCTA. Positive findings prompt appropriate operative, endovascular intervention or further invasive investigation with traditional modalities (esophagram/esophagoscopy and bronchoscopy) in those with potential aerodigestive injury. In the presence of significant foreign body artifact or other technical limitation, or for studies that remain equivocal yet concerning, conventional angiography is employed.

## EVIDENCE SUPPORTING USE OF MDCTA

In a multi-center prospective trial of 453 patients with penetrating neck trauma, MDCTA achieved 100% sensitivity and 97.5% specificity for the detection of clinically significant injury when compared against an aggregate gold standard incorporating the results of surgical exploration, catheter-based angiography, bronchoscopy, esophagram and esophagoscopy results, and clinical follow-up.^3^ In a smaller, earlier single-center prospective study investigating all patients without “hard” signs with MDCTA, sensitivity of 100% and specificity of 93.5% were reported.^4^ Earlier studies reported the sensitivity of MDCTA for detection of cervical vascular injury to range from 90% to 100%, with specificity of 98.6%–100%, a positive predictive value of 92.8%–100%, and a negative predictive value of 98%–100% when compared against the gold standard of conventional angiography.^5^,^6^ Both retro-and prospective analyses have suggested MDCTA is effective at reducing the number of invasive tests required in patients with penetrating neck injury.^7^,^8^

## SUMMARY

Evaluation of the patient with penetrating neck trauma has traditionally relied primarily on physical exam findings and assessment of patient hemodynamics, in association with selective multi-modal, invasive investigation to rule out vascular or aerodigestive injury in clinically stable patients. Although an effective strategy, and much superior to previous policies of routine exploration for penetrating injury, this approach still relies heavily on resource-intensive and invasive exams. A protocol-driven approach, integrating MDCTA with physical exam findings, and the clinical distinction of “hard” signs, “soft” signs, and “no” signs of vascular or aerodigestive injury, minimizes both the rate of negative explorations and the need for further invasive testing, decreasing overall resource burden and preventing unnecessary patient morbidity. Patients with hard signs proceed directly to the operating room. Completely asymptomatic patients may be observed. In those with soft signs, initial screening with MDCTA has high sensitivity for vascular injury and allows risk stratification of patients with possible aerodigestive trauma for further diagnostic investigation or intervention. MDCTA should be the first-line test in the evaluation of these patients. Patients with negative MDCTAs can be safely observed and discharged. Clinically stable patients with equivocal or concerning MDCTA findings should undergo appropriate further evaluation with traditional angiography, contrast studies, or endoscopy.

## Abbreviations:

ATLS: advanced trauma life support
MDCTA: multidetector computed tomographic angiography
